# Supplementary material for: Antibiofilm Activity and Mechanism of Linalool against Food Spoilage Bacillus amyloliquefaciens
Source: Int J Mol Sci. 2023 Jul 1;24(13):10980. doi: 10.3390/ijms241310980 (PMC10341759; doi:10.3390/ijms241310980)
Supplement: Supplementary file 1 [file ijms-24-10980-s001.zip › ijms-2444871-supplementary.pdf]

Table S1 Molecular docking results of linalool binned with potential targets.

No.	Receptor	Affinity kcal/mol)	Number of conventional H-bonds	Closest interacting residues	Distance to closest interacting residue (Å)
1	AbrB	-4.8	0	Met1, Lys2, Ser3	Lys2(3.39)
2	CodY	-4.6	0	Met1, Ala2, Leu3, Gln5, Lys6	Met1(2.71)
3	ComER	-4.7	0	Lys2, Ile3, Ala24, His29, Asp143, Thr146, Leu147, Glu64, Ile65	Met1(3.06)
4	ComP	-6.4	0	Met1, Arg2, Tyr3, Phe4, Ile5, Lys6, Gly334, Arg337	Tyr3(2.92)
5	ComQ	-4.8	0	Met1, Cys2, Leu3, Asp4, Phe46, Val50, Leu290	Cys2(3.36)
6	ComX	-4.9	1	Met1, Cys2, Leu3, Asp4, Phe46, Leu290	Cys2(2.49)
7	CwlO	-4.7	0	Met1, Phe2, Ile3, Ile4, Glu5, Gly29, Val33, Gln292, Glu296	Met1(3.31)
8	CysL	-5.4	0	Met1, Tyr2, Glu5, Leu6, Asn38, Leu39, Glu42	Met1(2.60)
9	DegQ	-3.8	0	Met1, Glu2, Lys3, Lys4, Leu5, Glu6	Met1(3.01)
10	DegU	-4.4	0	Met1, Thr2, Lys3	Met1(2.55)
11	EpsB	-5.0	0	Met1, Gly2, Phe3, Arg4, Lys215, Lys216, Lys217, Ile218, Lys219	Lys217(2.89)
12	epsG	-5.1	0	Met1, Ile2, Val3, Tyr4, Val5, Val6, Val56, Tyr147, Pro353, Ser356, Arg362, Thr363	Met1(2.63)
13	FabF	-5.2	0	Met1, Ser2, Lys3	Ser2(3.25)
14	FabHA	-5.6	0	Met1, Lys2, Ala3, Glu127, Gln129, Pro168, Val169, Ser170, Asp171	Lys2(3.26)
15	FabHB	-5.2	0	Met1, Ser2, Lys3, Asp170, Glu171	Met1(4.13)
16	FabI	-5.1	0	Met1, Asn2, Phe3	Met1(3.43)
17	FabL	-5.3	0	Met1, Glu2, Gln3, Lys27, Tyr29, Ser229, Ser230	Lys27(3.31)
18	FbpC	-5.2	0	Met1, Phe2, Trp3, Leu4, Phe5, Ile6, Trp69, Tyr134	Leu4(3.26)
19	FlgE	-5.1	0	Met1, Leu2, Arg3, Ser4, Leu5, Tyr6, Ser246, Leu250	Met1(3.49)
20	FtsE	-5.2	0	Met1, Ile2, Glu3, Met24, Ile25, His26, Pro27, Glu29, Val31, Leu60, Ile61, Asn62, His63, Arg206	Met1(2.77)
21	GalE	-5.2	1	Met1, Ala2, Ile3, Gly24, Tyr25, Glu26, Glu75	Ala2(2.23)

22	GapB	-4.5	0	Met1, Lys2, Val3, Gln26, Pro337, Ser338, Val340	Met1(3.19)
23	GtaB	-5.5	0	Met1, Lys2, Lys3, Leu153	Met1(2.27)
24	Hag	-4.3	1	Met1, Arg2, Ile3, Asn4, His5, Leu326	Asn4(3.04)
25	Icd	-5.2	0	Met1, Ser2, Gln3, Ile21, Thr57, Trp58, Lys59, Glu60,	Lys59(4.43)
26	KinC	-5.3	0	Met1, Gly2, Gln5, Ala6, Ile9, Lys59	Met1(2.35)
27	KinD	-5.6	0	Met1, Asn2, Thr3, Phe4	Met1(3.22)
28	LeuA	-6.0	1	Met1, Ser2, Asn3, Lys4, Ala5	Met1(3.23)
29	LuxS	-3.9	1	Met1, Pro2, Ser3, Glu8	Met1(2.20)
30	MalP	-5.2	0	Met1, Met2, Gln3, Lys4, Val5, Gln6	Met1(3.15)
31	MecA	-5.0	0	Met1, Trp2, Thr3, Glu4, Trp5	Met1(2.97)
32	MstX	-4.5	0	Met1, Lys2, Val3	Met1(2.41)
33	PhrC	-3.9	0	Met1, Lys2, Leu3, Lys4, Ser5	Met1(2.45)
34	PrmC	-5.4	0	Met1, Lys2, Thr3, Pro47, Val48, Leu53	Lys2(3.87)
35	PtkA	-4.8	0	Met1, Leu2, Thr3, Pro47, Gly187	Leu2(3.85)
36	PyrG	-4.6	0	Met1, Thr2, Lys3, Asp136, Asn169, Met265, Lys266, Leu267, Glu268	Leu267(3.33)
37	RapA	-5.5	0	Met1, Lys2, Gln3, Thr4	Lys2(3.30)
38	RapC	-5.6	0	Met1, Lys2, Ser3	Met1(3.57)
39	ResD	-4.7	0	Met1, Asp2, Gln3, Thr4, Asn5	Met1(2.81)
40	ResE	-4.9	2	Lys1, Phe2, Trp3, Lys4, Ser5, Gly8	Trp3(3.63)
41	SdhA	-5.5	0	Met1, Ser2, Gln3, Glu418	Met1(3.16)
42	SinI	-4.6	0	Met1, Lys2, Asn3	Met1(2.85)
43	sinR	-5.3	0	Met1, Thr2, Leu3, Ile4, Gly5, Asn39, Thr42, Leu63, Leu64, Asp65, Lys67	Met1(2.29)
44	SipW	-4.8	0	Met1, Lys2, Lys3, Thr4, Leu5, Lys6	Met1(2.80)
45	SlrR	-5.2	0	Met1, Ile2, Gly3, Arg4, Ile5, Leu61, Phe62, Ala64, Glu65, Thr66, Met67, His69	Ile2(2.72)
46	SpeB	-4.7	0	Met1, Arg2, Phe3, Asp4, Glu5, Ala6, Ala14, Arg16, Ala67	Met1(2.82)
47	Spo0F	-4.5	0	Met1, Met2, Asn3, Lys122	Met1(2.94)
48	SpoVG	-4.7	0	Met1, Glu2, Val3, Leu25, Asp26, Thr68, Lys71	Met1(3.36)
49	TapA	-4.9	0	Met1, Phe2, Arg3	Phe2(2.37)
50	TasA	-6.3	2	Met1, Gly2, Met3, Lys4, Lys5, Lys6, Asp36, Leu57, Ser58, Asn59, Lys93, Asn220, Ala221, Ile222, Ser223	Met1(2.51)
51	ThrB	-5.0	0	Met1, Asn2, Glu3, Glu5	Met1(2.87)

52	TrxA	-5.5	0	Met1, Ser2, Tyr390, Gln399, Asp420, Leu422, Gln423	Met1(3.70)
53	Veg	-4.5	0	Met1, Ala2, Lys3	Ala2(2.41)
54	YaaB	-4.3	0	Met1, Tyr2, Ile3, Ser12, Thr13, Arg14, Met76	Met1(3.06)
55	Ydak	-4.7	0	Met1, Lys2, Ile3, Ser4, Phe5, Asn6, Ser119, Leu123	Met1(2.20)
56	YlzA	-3.9	0	Met1, Thr2, Ile3	Met1(2.83)
57	YmcA	-4.5	0	Met1, Thr2, Leu3	Met1(2.75)
58	YmdB	-4.8	0	Met1, Arg2, Ile3, His32, Asn106, Lys108, Tyr144, Ile239, Asp240, Ile241, Asp242, Asp243, Gln244	Met1(3.31)
59	YpqP	-4.8	0	Met1, Gly2, Ala3, Thr4, Lys5, Leu6	Met1(3.35)
60	YtrA	-4.3	0	Met1, Ile2, Leu23, Ile28, Pro36, Leu41, Ile44, Ile45	Met1(2.80)
61	YtrB	-5.0	0	Met1, Ile2, Glu3, Thr23, Ile24, Gly25, Lys26, Glu28, Leu59, Phe60, Asn61, Gln62, Arg196	Ile24(3.03)
62	YtrC1	-5.3	0	Met1, Val2, Gly3, Leu4, Phe5, Ile53, Leu54, Val92, Phe96	Met1(2.48)
63	YtrC2	-5.4	0	Met1, Ile2, Gln103, Tyr248	Met1(2.58)
64	YtrD	-6.2	0	Met1, Asn2, Met3, Phe4, Gly15, Tyr18	Met1(4.18)
65	YtrE	-5.2	1	Met1, Ile2, Asp3, Thr31, Val32, Glu33, Lys34, Val67, Ile68, Asn69, Gly70	Glu33(3.14)
66	YtrF	-4.8	0	Met1, Arg2, Phe3, Asp5, Gln6, Ile339	Met1(3.68)
67	YugO	-5.1	0	Met1, Lys2, Ser3, Arg5	Met1(4.05)
